# Long-term effect of chronic hepatitis B on mortality in HIV-infected persons in a differential HBV transmission setting

**DOI:** 10.1186/s12879-022-07477-1

**Published:** 2022-05-27

**Authors:** Justine Umutesi, Sabin Nsanzimana, Carol Yingkai Liu, Patrizio Vanella, Jördis J. Ott, Gérard Krause

**Affiliations:** 1 Helmholtz Centre for Infection Research (HZI)-PhD Program “Epidemiology”, Brunswick, Germany; 2 Department of Epidemiology, Helmholtz-Zentrum Für Infektionsforschung GmbH (4214), Inhoffenstr. 7, 38124 Brunswick, Germany; 3 Rwanda Biomedical Center, Kigali, Rwanda; 4 Department of Epidemiology, Emory University Rollins School of Public Health, Atlanta, USA; 5 Medizinische Hochschule Hannover (3118), Hannover, Germany; 6 University of Rostock, Rostock, Germany; 7 TWINCORE, Zentrum für Experimentelle und Klinische Infektionsforschung GmbH (8925), Hannover, Germany; 8 German Center for Infection Research (DZIF), Cologne, Germany

**Keywords:** Chronic hepatitis B, Mortality, HIV, Cohort study, Sub-Saharan Africa, Rwanda

## Abstract

**Background:**

There remain gaps in quantifying mortality risk among individuals co-infected with chronic hepatitis B (HBV) and human immunodeficiency virus (HIV) in sub-Saharan African contexts. Among a cohort of HIV-positive individuals in Rwanda, we estimate the difference in time-to mortality between HBV-positive (HIV/HBV co-infected) and HBV-negative (HIV mono-infected) individuals.

**Methods:**

Using a dataset of HIV-infected adults screened for hepatitis B surface antigen (HBsAg) from January to June 2016 in Rwanda, we performed time-to-event analysis from the date of HBsAg results until death or end of study (31 December 2019). We used the Kaplan–Meier method to estimate probability of survival over time and Cox proportional hazard models to adjust for other factors associated with mortality.

**Results:**

Of 21,105 available entries, 18,459 (87.5%) met the inclusion criteria. Mean age was 42.3 years (SD = 11.4) and 394 (2.1%) died during follow-up (mortality rate = 45.7 per 100,000 person-months, 95% confidence interval (CI) 41.4–50.4) Mortality rate ratio for co-infection was 1.7, 95% CI 1.1–2.6, however, Cox regression analysis did not show any association with mortality between compared groups. The adjusted analysis of covariates stratified by co-infection status showed that males, residing outside of the capital Kigali, drinking alcohol, WHO-HIV-clinical stage 3 and 4 were associated with increased mortality in this HIV cohort.

**Conclusions:**

HBV infection does not significantly influence mortality among HIV-infected individuals in Rwanda. The current cohort is likely to have survived a period of high-risk exposure to HBV and HIV mortality and limited health care until their diagnosis.

**Supplementary Information:**

The online version contains supplementary material available at 10.1186/s12879-022-07477-1.

## Background

Globally in 2020, 820,000 individuals died from chronic hepatitis B virus infection (HBV), compared to 680,000 people dying from HIV disease [1], and approximately 2.6 million HIV-infected individuals are co-infected with HBV [2]. Although HIV and HBV can share similar transmission routes [3], the contribution of transmission routes to infection burden may differ by geographical region [2]. For example, studies in East Africa have shown that scarification and blood transfusions are important risk factors for HBV transmission [4] and that HBV is often acquired at a younger age (before 5 years) compared to other regions in Africa. In contrast, HIV transmission in East Africa is driven by heterosexual sex in adulthood [2]. Low levels of HIV transmission among younger age groups can be explained by the recent introduction of antivirals in Africa [5] for prevention of mother-to-child transmission. The pathophysiology of HBV and the duration of the infection in East Africa, is an important factor to suspect a different risk of mortality that is assumed to be different of that in developed countries where, advanced health infrastructure for vaccination, screening and treatment make HBV acquisition in adulthood through sex and injecting drug use [6].

Several studies have found reduced survival [7–10] of HBV and HIV co-infected individuals, while other large studies [11–13] found no effect. These discrepancies, along with the potential lack of generalizability of previous studies for the eastern African population, raise reasonable question on the effect of HIV–HBV co-infection on mortality risk, warranting local-level investigation for more targeted policy guidance.

We conducted this study in Rwanda, an East African country where identification of people with HBV–HIV co-infection began in 2016 after a long period of uncertainty about co-infection and its impact on population mortality. Building on the previous work by Umutesi et al. (2021), we focus on HIV-infected people, and take into account the introduction of universal, early and immediate antiretroviral therapy (ART) [14] that raised ART coverage to 97.5% in Rwanda [15], and the prioritization of TDF as a first-line treatment [16]. There is little evidence of the effect of HBV on mortality in HIV-infected individuals, particularly in large cohorts with routine HIV care, routine free HIV/HBV testing, high antiretroviral treatment coverage and in the context of a differential HBV transmission route [2]. Using a large HIV-infected individual cohort in sub Saharan Africa, we examined the association between chronic HBV infection and HIV mortality among HIV-infected individuals.

## Methods

### Study design, population and settings

In this retrospective cohort study, we use data derived from a nationwide cohort of 117,258 HIV-infected adults (15 years and above) screened for hepatitis B surface antigens (HBsAg) during a vaccination and screening campaign in 2016 (the characteristics of this population is presented in Additional file 2: Appendix S2). The set-up, process and other details of this campaign have been described by Umutesi et al. [17]. In addition, a national electronic medical record (EMR) registry was used to supplement individual clinical, immunological and treatment data. The information on the Rwanda EMR has been published in earlier studies [18, 19]. The study period was from 1 January 2016 to 31 December 2019, included 21,105 records from populations with available HBV test results available between January and February 2016, and have been registered in one of the 340 HIV clinics with an EMR system in 2016.

### Data processing and data collection

Data were collected using a predefined list of variables. This was developed and approved by the study committee after consultation of the literature on factors affecting survival of HIV-infected individuals. The unique patient identifier in the HBV vaccination and screening database and in the national EMR database for HIV-infected patients were matched, as the EMR did not contain HBV results. The HBV vaccination and screening database showed that as of 31 January 2016, HBV results were available for 59 health facilities and only 40 sites had EMR backups. To include all provinces, urban and rural district hospitals and health centers, we extended the period to 29 February 2016, and 62 of the 71 facilities including six district hospitals and 56 health centers were included and were visited to manually complete missing data, which resulted in 21,105 records for analysis. We considered these data sufficient to cover residence and geographic characteristics. Health care personnel involved in recording patient data received constant supervision and mentoring to ensure high quality data. In addition, data found to be incomplete or inconsistent were cross-checked on-site with patient files and patient registers. The principles of data protection and good scientific practice were strictly followed, both in Rwanda and in Germany, where the analysis was conducted and ethics committees in both countries approved the study (No. 958/RNEC/2019 and Nr.8604_BO_K_2019).

### Measurement and outcome definitions

We assessed all-cause mortality, defined as death from any cause during the study period, as medically recorded in the EMR. The follow-up period was disaggregated into months from 1 January 2016 to 31 December 2019. Two groups were compared: HBsAg-positive participants, defined as co-infected with HBV and HIV, and HBsAg-negative participants, defined as HIV mono-infected individuals. Independent variables included age, disaggregated into four groups: 15–34, 35–54, 55–64 and 65+, sex categorized as less than and more than 7 years. Province of residence, TDF or non-TDF-based regimen defined as the last registered antiretroviral treatment containing TDF or not otherwise, HIV clinical staging according to WHO guidelines [20]. Diabetes, history of tuberculosis, duration of HIV treatment and time since HIV testing which were classified into two categories: less than 7 years and 7 years and over. Adherence to treatment measured as good when recorded as 95% and above and low when recorded as below 95% [21]. Biological factors, such as CD4 cell count at HBsAg testing binarized into low when below 350 cells/mm^3^ and high when 350 cells/mm^3^. HIV viral load (VL) at HBsAg testing considered to be undetectable when below 1000 copies/ml and detectable when above 1000 copies/ml [22]. Socio-behavioral factors, such as smoking history and alcohol use, were also assessed as binary yes/no information.

### Statistical analysis

For each variable analyzed, we computed the prevalence of HBsAg and the mortality rate. Each participant was followed from the date of HBsAg test result and censored at death or at administrative censoring on December 31, 2019. Missing variables (> 5%) were not analyzed unless the variable is considered an important factor of mortality in HIV infected individuals. Data on transfer-out and lost to follow-up were considered as incomplete as there was no further information provided and were removed from the main analysis. However, we described them separately by co-infection category for different covariates to assess whether they might have had any impact on our results, and the results have been presented in Additional file 3: Appendix S3. In addition, individuals with no HBsAg results and individuals with positive hepatitis C virus (HCV) antibody results were removed to avoid confounding and because chronic HCV infections could not be determined. We calculated mortality rates by dividing the number of deaths by the person-months of follow-up and survival time (in months) by subtracting the date of HBsAg testing results from the date of death for both co-infected and mono-infected individuals. We used a non-parametric Kaplan–Meier survival modeling [23] to visualize overall survival experience of the entire cohort, stratified by chronic HBV co-infection status, and then by selected covariates known to be associated with mortality. In addition to visualization, we tested the difference between the survival curves by comparing the survival probability of the covariates using the log-rank test (given an equal chance at each time point). We used the Cox proportional hazards model to estimate multivariable adjusted hazard ratios (or ratios of mortality rates between study groups) and their 95% confidence intervals (CIs). To allow a flexible and a wide effect model, during variable selection for model inclusion, we considered variables that had a p-value of 0.10 and below in unadjusted regression models and all possible interactions. Modeling was performed using stepwise backwards deletion, assessing the magnitude of change in effect of the covariates for variable retention. Adjustments were made for sex, age, province of residence, smoking habit, alcohol use, CD4 cells levels, HIV VL copies, TDF based regimen, ART adherence, WHO clinical staging of HIV/AIDS, tuberculosis, time from HIV testing and time from ART initiation. The proportional hazard assumption of the risk function was tested using the Schoenfeld test [24]. We presented unadjusted and adjusted hazard ratios and their 95% CIs and assessed the statistical significance with a two-sided alpha of 0.05.

## Results

### Flow diagram of the study participants

**Fig. 1:**
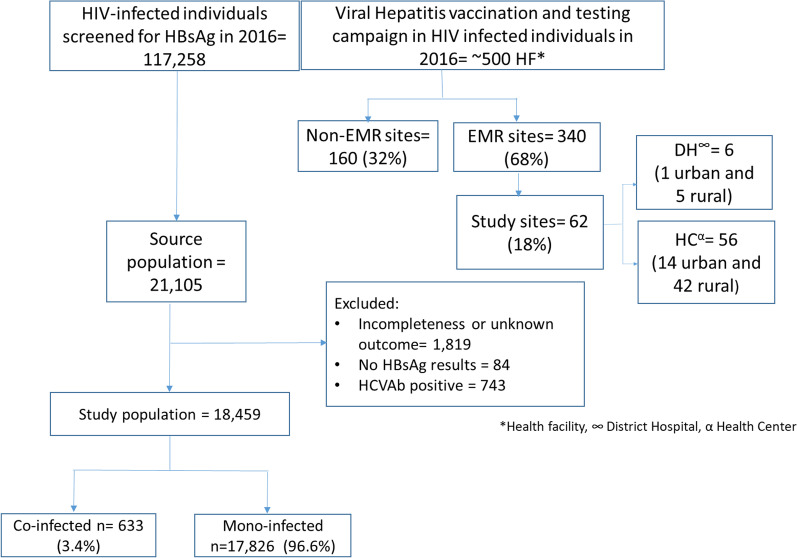
Study population and sites

Figure 1 shows that of a total of 117,258 HIV-infected individuals who participated in the 2017 vaccination and screening campaign, 21,105 from 62 health facilities met the study criteria and were therefore included in the study, with the details of their selection explained earlier in “Methods” section.

### Characteristics of study participants

The study population were followed from the date of HBsAg results to death or end of the study period. Table 1 presents the characteristics of the study population using median and interquartile range (IQR) or mean and standard deviation (SD) for continuous covariates, or as frequencies and percentages for categorical data and differences in distribution among co-infected and mono-infected were assessed using the Pearson Chi-square test [25]. The study population had a mean age of 42.3 years (SD 11.4) and 65.1% were women. The prevalence of HBV infection by groups is shown in Additional file 1: Appendix S1.

**Table 1:** Characteristics of study population

Covariates	Category	Overall frequency [n = 18,459 (%)]	HIV mono-infected [n = 17,826 (%)]	HIV–HBV co-infected [n = 633 (%)]
Sex	Female	12,009 (65.1)	11,669 (65.5)	340 (53.7)
	Male	6424 (34.8)	6133 (34.4)	291 (46.0)
	*Unknown*	26 (0.1)	24 (0.1)	2 (0.3)
Age	Mean (SD)	42.3 (11.4)	42.3 (11.4)	41.8 (10.6)
	15–24	1131 (6.1)	1098 (6.2)	33 (5.2)
	25–34	3520 (19.1)	3394 (19.0)	126 (19.9)
	35–44	5962 (32.3)	5744 (32.2)	218 (34.4)
	45–54	5188 (28.1)	4998 (28.0)	190 (30.0)
	55–64	2069 (11.2)	2011 (11.3)	58 (9.2)
	65+	470 (2.5)	464 (2.6)	6 (0.9)
	*Unknown*	119 (0.6)	117 (0.7)	2 (0.3)
Province	City of Kigali	6660 (36.1)	6354 (35.6)	306 (48.3)
	East	329 (1.8)	321 (1.8)	8 (1.3)
	North	3601 (19.5)	3477 (19.5)	124 (19.6)
	South	2804 (15.2)	2739 (15.4)	65 (10.3)
	West	5065 (27.4)	4935 (27.7)	130 (20.5)
Current or past smokers	No	17,953 (97.3)	17,341 (97.3)	612 (96.7)
	Yes	428 (2.3)	414 (2.3)	14 (2.2)
	*Unknown*	78 (0.4)	71 (0.4)	7 (1.1)
Current or former drinker	No	14,130 (76.5)	14,056 (78.9)	74 (11.7)
	Yes	4243 (23.0)	3691 (20.7)	552 (87.2)
	*Unknown*	86 (0.5)	79 (0.4)	7 (1.1)
CD4 (cells/mm^3^) at HBsAg testing	Median (IQR)	345 (168–564)	346 (168–564)	322 (162–544)
	< 350	2,965 (16.1)	2,857 (16.0)	108 (17.1)
	≥ 350	4,446 (24.1)	4,307 (24.2)	139 (22.0)
	*Unknown*	11,048 (59.9)	10,662 (59.8)	386 (61.0)
HIV viral load (copies/ml)	Median (IQR)	20 (20–20)	20 (20–20)	20 (20–20)
	< 1000	12,234 (66.3)	11,838 (66.4)	396 (62.6)
	≥ 1000	480 (2.6)	460 (2.6)	20 (3.2)
	*Unknown*	5745 (31.1)	5528 (31.0)	217 (34.3)
ART adherence	Bad (< 95%)	1067 (5.8)	1050 (5.9)	17 (2.7)
	Good (≥ 95%)	16,380 (88.7)	15,815 (88.7)	565 (89.3)
	*Unknown*	1012 (5.5)	961 (5.4)	51 (8.1)
WHO stage	1&2	13,909 (75.4)	13,472 (75.6)	437 (69.0)
	3&4	3900 (21.1)	3737 (21.0)	163 (25.8)
	*Unknown*	650 (3.5)	617 (3.5)	33 (5.2)
Tuberculosis	No	17,701 (95.9)	17,087 (95.9)	614 (97.0)
	Yes	758 (4.1)	739 (4.1)	19 (3.0)
Diabetes	No	17,947 (97.2)	17,336 (97.3)	611 (96.5)
	Yes	54 (0.3)	53 (0.3)	1 (0.2)
	*Unknown*	458 (2.5)	437 (2.5)	21 (3.3)
TDF based regimen	No	4608 (25.0)	4454 (25.0)	154 (24.3)
	Yes	13,339 (72.3)	12,887 (72.3)	452 (71.4)
	*Unknown*	512 (2.8)	485 (2.7)	27 (4.3)
Time since HIV testing	< 7 years	7227 (39.2)	6971 (39.1)	256 (40.4)
	≥ 7 years	8960 (48.5)	8670 (48.6)	290 (45.8)
	*Unknown*	2272 (12.3)	2185 (12.3)	87 (13.7)
Time since ART start	< 7 years	11,846 (64.2)	11,460 (64.3)	386 (60.0)
	≥ 7 years	6300 (34.1)	6071 (34.1)	229 (36.2)
	*Unknown*	313 (1.7)	295 (1.7)	18 (2.8)

### Mortality rates by characteristics of the study population

The overall mortality rate was 45.7 per 100,000 person-months, with co-infected individuals, men and the oldest age group of 65 years and above experiencing a higher mortality rate as presented in Table 2.

**Table 2:** Mortality rates per 100,000 person months

Covariates	Category	Overall frequency (%) (n = 18,459)	Number of deaths, n = 394 (2.1%)	Person-months of follow-up = 862,152	Mortality rates per 100,000 person-months (95% CIs) 45.7 (41.4–50.4)
HBsAg	Negative	17,826 (96.6)	371 (2.1)	832,495	44.5 (40.2–49.3)
	Positive	633 (3.4)	23 (3.6)	29,657	77.5 (51.5–116.7)
Sex	Female	12,009 (65.1)	190 (1.6)	560,010	33.9 (29.4–39.1)
	Male	6424 (34.8)	204 (3.2)	300,907	67.8 (59.1–77.8)
	*Unknown*	26 (0.1)			
Age	Mean (SD)	42.3 (11.4)			
	15–24	1131 (6.1)	19 (1.7)	52,972	35.9 (22.9–56.2)
	25–34	3520 (19.1)	49 (1.4)	164,656	29.7 (22.5–39.4)
	35–44	5962 (32.3)	112 (1.9)	278,602	40.2 (33.4–48.4)
	45–54	5188 (28.1)	107 (2.1)	242,476	44.1 (36.5–53.3)
	55–64	2069 (11.2)	66 (3.2)	96,390	68.4 (53.8–87.1)
	65+	470 (2.5)	39 (8.3)	21,481	181.5 (132.6–248.5)
	*Unknown*	119 (0.6)	2 (1.7)		
Province	City of Kigali	6660 (36.1)	98 (1.5)	311,615	31.5 (25.8–38.3)
	East	329 (1.8)	3 (0.9)	15,583	19.2 (6.2–59.7)
	North	3601 (19.5)	104 (2.9)	166,030	62.6 (51.7–75.9)
	South	2804 (15.2)	59 (2.1)	130,224	45.3 (35.1–58.5)
	West	5065 (27.4)	130 (2.6)	238,699	54.5 (44.9–64.7)
Current or past smokers	No	17,953 (97.3)	368 (2.0)	838,705	43.9 (39.6–48.6)
	Yes	428 (2.3)	22 (5.1)	19,869	110.7 (72.9–168.1)
	*Unknown*	78 (0.4)	4 (5.1)		
Current or former drinker	No	14,130 (76.5)	155 (1.1)	662,049	23.4 (20.0–27.4)
	Yes	4243 (23.0)	230 (5.4)	196,209	117.2 (103.0–133.4)
	*Unknown*	86 (0.5)	9 (10.5)		
CD4 (cells/mm^3^) at HBsAg testing	Median (IQR)	345 (168–564)			
	< 350	2965 (16.1)	63 (2.1)	138,533	45.5 (35.5–58.2)
	≥ 350	4446 (24.1)	94 (2.1)	207,634	44.3 (37.0–55.4)
	*Unknown*	11,048 (59.9)	237 (2.1)		
HIV viral load (copies/ml)	Median (IQR)	20 (20–20)			
	< 1000	12,234 (66.3)	205 (1.7)	573,153	35.8 (31.2–41.0)
	≥ 1000	480 (2.6)	13 (2.7)	22,385	58.1 (33.7–100.0)
	*Unknown*	5745 (31.1)	176 (3.1)		
ART adherence	Bad (< 95%)	1067 (5.8)	16 (1.5)	49,867	32.1 (19.7–52.4)
	Good (≥ 95%)	16,380 (88.7)	232 (1.4)	767,465	30.2 (26.6–34.4)
	*Unknown*	1012 (5.5)	146 (14.4)		
WHO-HIV clinical stage	1&2	13,909 (75.4)	217 (1.6)	651,325	33.3 (29.2–38.1)
	3&4	3900 (21.1)	112 (2.9)	181,345	61.8 (51.3–74.3)
	*Unknown*	650 (3.5)	65 (10.0)		
Tuberculosis	No	17,701 (95.9)	364 (2.1)	826,936	44.0 (39.7–48.8)
	Yes	758 (4.1)	30 (4.0)	35,215	85.2 (59.6–121.8)
Diabetes	No	17,947 (97.2)	373 (2.1)	838,394	44.5 (40.2–49.2)
	Yes	54 (0.3)	0	2542	0
	*Unknown*	458 (2.5)	21 (4.6)		
TDF based regimen	No	4608 (25.0)	99 (2.1)	215,216	46.0 (37.8–56.0)
	Yes	13,339 (72.3)	273 (2.0)	623,266	43.8 (38.9–49.3)
	*Unknown*	512 (2.8)	22 (4.3)		
Time since HIV testing	< 7 years	7227 (39.2)	177 (2.4)	337,736	52.4 (45.2–60.7)
	≥ 7 years	8960 (48.5)	135 (1.5)	419,021	32.2 (27.2–38.1)
	*Unknown*	2272 (12.3)	82 (3.6)		
Time since ART start	< 7 years	11,846 (64.2)	266 (2.2)	553,332	48.1 (42.6–54.2)
	≥ 7 years	6,300 (34.1)	94 (1.5)	294,636	31.9 (26.1–39.0)
	*Unknown*	313 (1.7)	34 (10.9)		

### Survival experience of participants according to hepatitis B co-infection

**Fig. 2:**
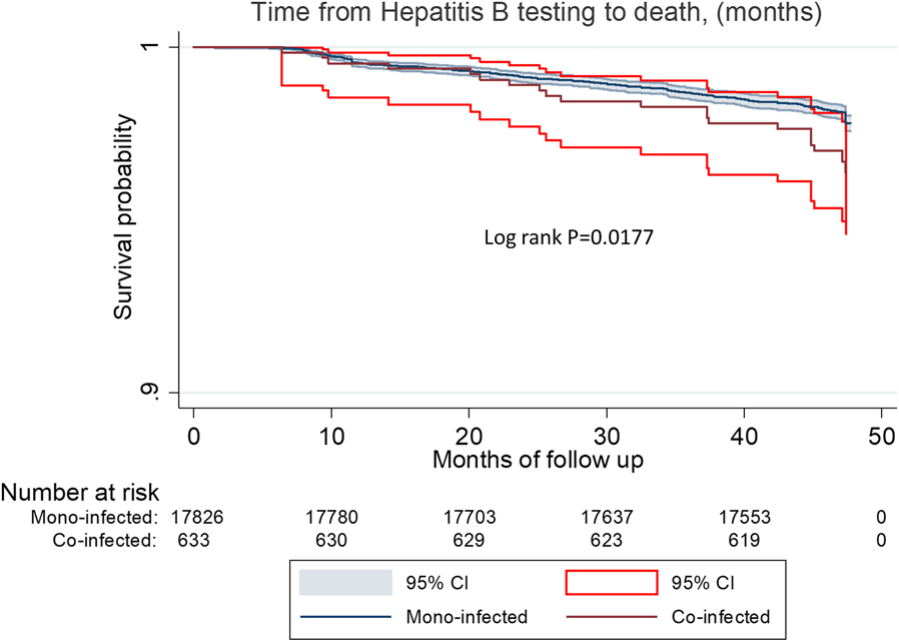
Kaplan–Meier curves showing mortality in HIV-infected individuals, by chronic HBV co-infection status

The Kaplan Meier curve (Fig. 2) shows the estimated survival probabilities by infection category during the follow-up period predominated by co-infected individuals.

Using log-rank test, crude analysis shows a statistically significant difference in the probability of survival between HIV mono-infected individuals and HIV/HBV co-infected individuals (p = 0.0117) as shown in Fig. 2.

### Multiple Cox regression analysis

The median time to mortality was 47.4 months (range 1.5–47.7 months). Table 3 shows the results of the univariate and multiple survival model. Crude analysis shows a statistically significant association of co-infection and time to mortality. All covariates included in the analysis-co-infection status, sex, age, residence, alcohol use, WHO-HIV stages, years since initiation of ART, smoking, history of tuberculosis, and TDF use-were statistically significant, except for TDF use, and were included in the Cox regression model. However, the adjusted analysis failed to identify an association with time to mortality between co-infected and mono-infected groups: adjusted hazard ratio (aHR) of 0.84 and a 95% CI of 0.52–1.36. Multiple Cox regression analysis of covariates stratified by co-infection status shows that among 18,216 (94.9%) observations retained for multiple Cox regression analysis, sex, and age, province of residence, drinking alcohol, WHO clinical staging of HIV/AIDS and time from ART initiation were predictive mortality in both crude and adjusted analysis. For example, compared to females, and holding other covariates constant, males had a 1.39 times higher aHR and the difference was statistically significant. Compared to people residing in the capital city, Kigali, those residing in the northern, southern and western province had a higher aHR. Our results show that individuals who initiated ART at least 7 years prior to their HBV diagnosis had lower mortality compared to those on ART for lesser than 7 years, Table 3.

**Table 3:** Unadjusted and adjusted Cox regression analysis of factors associated with survival stratified by HBV coinfection status

Covariates	Category	CHR^a^ (95% CIs)	p-value^b^	AHR^c^ (95% CIs)	p-value^b^
HBsAg results (ref. positive)	Positive	1.65 (1.08–2.52)	0.019	0.84 (0.52–1.36)	0.486
Sex (ref. Female)	Male	1.87 (1.53–2.289)	< 0.001	1.39 (1.11–1.76)	0.005
Age (ref. 15–24)	25–34	0.84 (0.49–1.42)	0.512	1.00 (0.54–1.83)	0.991
	35–44	1.13 (0.69–1.83)	0.631	1.12 (0.64–1.97)	0.695
	45–54	1.22 (0.75–2.00)	0.413	1.13 (0.64–2.00)	0.671
	55–64	1.90 (1.14–3.16)	0.014	1.66 (0.91–3.01)	0.096
	65+	5.07 (2.93–8.77)	< 0.001	3.41 (1.80–6.49)	< 0.001
Province (ref. Kigali City)	East	0.58 (0.18–1.83)	0.352	0.53 (0.13–2.15)	0.373
	North	2.07 (1.57–2.72)	< 0.001	2.03 (1.49–2.78)	< 0.001
	South	1.47 (1.06–2.03)	0.019	1.57 (1.10–2.24)	0.013
	West	1.64 (1.26–2.14)	< 0.001	1.55 (1.14–2.09)	0.005
Current or former drinker (ref. No)	Yes	3.92 (4.01–6.03)	< 0.001	4.20 (3.32–5.31)	< 0.001
WHO stages (ref. 1&2nd stage)	3&4 stage	1.85 (1.47–2.32)	< 0.001	2.15 (1.68–2.75)	< 0.001
Time since ART initiation (ref. < 7 years)	≥ 7 years	0.66 (0.52–0.84)	0.001	0.53 (0.41–0.70)	< 0.001
Ever smoked (ref. No)	Yes	2.49 (1.62–3.83)	< 0.001	0.92 (0.57–1.49)	0.736
Ever had tuberculosis (ref. No)	Yes	1.92 (1.32–2.79)	0.001	1.33 (0.86–2.06)	0.200
TDF based regimen (ref. No)	Yes	0.95 (0.76–1.20)	0.676		

^a^Crude hazard ratio, ^b^Chi squared p-value, ^c^adjusted hazard ratio

## Discussion

Our results show that chronic HBV co-infection is not associated with time to mortality in HIV-infected individuals in Rwanda. We consider the following are reasons for minimal effect of HBV–HIV co-infection on increased mortality. First, by the end of 2007, about 10 years before participants in the current study could be tested for HBV, 80% of eligible HIV-positive Rwandans were on ART [26] a primary reason for a possible reduction in overall HIV-related mortality in Rwanda. We further note that 17,947/18,459 (97.2%) of our study population were on highly active antiretroviral therapy (HAART), and of these 13,339 (72.3%) were on a TDF-based regimen. In fact, TDF has been used in first-line ART in Rwanda since 2009 and various research studies have shown that it reduces HBV replication [27], thereby improving HIV outcomes, including mortality. Second, 100% of our population was on Lamividine (also known as 3TC, Epivir), another drug that reduces HBV replication [28]. Third, we consider our study population to be individuals who have succeeded or survived a past where the risk of infection and mortality was higher due to poor health infrastructure and insufficient knowledge of HIV and HBV in the population. In fact, African populations have carried a huge proportion of HBV infection for decades [29] and it is therefore indisputable that chronic HBV is a major risk factor for mortality in untreated individuals [30], and anti-HBV drugs have recently been introduced in African countries [31]. Consultation with traditional healers in the past is another reason that may increase the progression of the disease and the many deaths of HBV chronically infected individuals. Fourth, we suspect differences in mortality can be observed over longer periods of follow-up beyond the follow-up time after HBV diagnosis allowed by our study. Finally, our population displayed good clinical control of HIV infection as measured by CD4 cell counts, HIV RNA load, adherence to antiretroviral therapy, comorbidities including TB and WHO-HIV clinical stage. Nevertheless, a meta-analysis of studies conducted before the introduction of HAART found an increase in mortality among HBV–HIV co-infected individuals compared to mono-infected individuals [7]. However, all studies included in the review were from developed countries in Europe and the United States, and no studies were conducted in Africa. In contrast, a cohort study of HIV-infected persons enrolled between 1984 and 2003 in Greece found no impact of HBV co-infection on all-cause mortality. In another recent study conducted by Thornton [10], an increase in all-cause mortality following HBV and HIV co-infection was found in England. Higher mortality among co-infected individuals in developed countries may not translate to higher mortality in developing countries since the demographic distribution of co-infected individuals and access to prevention and treatment services between the two contexts is importantly different. For example, co-infected individuals from developed countries are more likely to be intravenous drug users and men who have sex with men, with potential differences in adherence to treatment contributing to higher mortality [10].

Although the prevalence of HBV/HIV co-infection is estimated to be over 28.4% in some African countries [32], a number of mortality studies have been conducted on specific populations. For example, a clinical trial conducted in Côte d’Ivoire by Kouamé in 2021 focused on people with low CD4 cell counts and found a higher impact of HIV and HBV co-infection on mortality [9]. However, in countries or regions where HAART is universal, this risk could be reduced, as in the case of an HIV clinic in Nigeria in 2014, where the study occurred before the implementation of TDF and HAART was a protective factor against mortality risk [12]. In the same vein, in countries where HAART was introduced in 1990s, such as in Europe [33], the effect of chronic HBV on liver-related mortality has been demonstrated but without any impact on progression to AIDS, immunological or virological response to HAART [34]. These scenarios could apply to Rwanda where, in 2020, 97.5% of HIV-infected persons were on HAART [15].

Independently to HBV co-infection status, our study showed that other factors increased the risk of mortality in HIV-infected persons, including being male. However, this finding is not consistently described in the literature where some have described a higher risk of mortality in women [35] while others a higher mortality risk among men [36, 37]. The increased case mortality risk in HIV-infected men may be related to a later diagnosis [38], later clinic attendance or lack of adherence to ART, as has been shown to be common among men in sub-Saharan Africa [39]. Age over 55 years versus 15–24 years and WHO-HIV stages 3 and 4 were predictive of mortality, which may be related to long-term HIV infection with more severe signs and symptoms or other comorbidities that, in turn, tend to increase with age [40]. The province of residence was also associated with the time to mortality, where residing in southern, northern and western province as compared to capital Kigali were highly associated with time to mortality. Previous studies have shown a higher risk of infection in provinces bordering neighboring countries [41] and a higher case fatality rate is likely. Other reasons could be related to health services centralized in Kigali and the increased likelihood of consulting traditional healers in certain peripheral regions of the county. Another predictor of mortality was alcohol consumption and its increased effect on liver disease was demonstrated [42, 43]. Finally, one co-variate, time since starting treatment, showed that taking ART for at least 7 years was associated with a lower risk of mortality, suggesting the effectiveness of ART [44].

Limitations of our study include a relatively short follow-up period that does not allow detection of a difference in mortality between the co-infected groups, because the development of cirrhosis and hepatocellular carcinoma (HCC) is too slow in patients on TDF [45]. Also the lack of quantification of the risk of HCCand liver-related mortality. As the data could not allow us to follow the study participants from the age of onset of HBV infection and that the immortal time bias is possible, we recommend further analysis to interpret the hazard ratio by age of acquisition of infection. We believe that prolonged duration between infection and diagnosis, an artefact of lack of prior health infrastructure, meaning a substantial proportion of this population died prior to the time diagnosis was offered and could not be included in our study. It is therefore unlikely that our results show a lethal effect of hepatitis B in the study population. We recognize the limitation of not being able to assess the actual survival time of those lost to follow-up, and lack of information on the completeness of EMR mortality data and other supporting sources, which may lead to reporting bias and thus conservative interpretation of the results of this study. Finally, considering that HBV viral load activity at the start of ART may also influence the difference in mortality [46, 47], we acknowledge that the lack of HBV DNA testing may limit our results.

## Supplementary Information

**Additional file 1: Appendix S1.** HBsAg prevalence by characteristics of the study population.

**Additional file 2: Appendix S2.** Comparing characteristics of all HIV infected screened in 2016 and the current study population.

**Additional file 3: Appendix S3.** Characteristics of individuals lost to follow up by co-infection categories.

## Data Availability

The data used during this study are not publicly available due to data protection regulation in Rwanda and Germany. However, upon reasonable request, the data can be obtained from the corresponding author: Justine.umutesi@helmholtz-hzi.de.
