# Supplementary material for: Long-term effect of chronic hepatitis B on mortality in HIV-infected persons in a differential HBV transmission setting
Source: BMC Infect Dis. 2022 May 27;22:500. doi: 10.1186/s12879-022-07477-1 (PMC9137150; doi:10.1186/s12879-022-07477-1)
Supplement: Supplementary file 1 — Additional file 1: Appendix S1. HBsAg prevalence by characteristics of the study population. [file 12879_2022_7477_MOESM1_ESM.docx]

**Appendix S1**

The overall prevalence of chronic HBV was 3.4% and HBsAg were more prevalent in men (p-value >0.001) with the highest prevalence found among people who drink or have drunk alcohol (13%).

Table 3 HBsAg prevalence by characteristics of the study population

| **Covariates** | **Category** | **HIV mono-infected (n=17,826)** | **HIV-HBV co-infected (N=633)** | **HBsAg positive (%)** | **p-value*** |
| --- | --- | --- | --- | --- | --- |
| **Overall n= 18,459** | | | | | |
| **HBsAg** | Negative | 17,826(96.6) |  |  | <0.001 |
|  | Positive |  | 633(3.4) |  |  |
| **Sex** | Female | 11,669(65.5) | 340(53.7) | 340(2.8) | <0.001 |
|  | Male | 6,133(34.4) | 291(46.0) | 291(4.5) |  |
|  | *Unknown* | 24(0.1) | 2(0.3) | 2(7.7) |  |
| **Age** | Mean (SD) | 42.3(11.4) | 41.8(10.6) |  | 0.036 |
|  | 15-24 | 1,098(6.2) | 33(5.2) | 33(2.9) |  |
|  | 25-34 | 3,394(19.0) | 126(19.9) | 126(3.6) |  |
|  | 35-44 | 5,744(32.2) | 218(34.4) | 218(3.7) |  |
|  | 45-54 | 4,998(28.0) | 190(30.0) | 190(3.7) |  |
|  | 55-64 | 2,011(11.3) | 58(9.2) | 58(2.8) |  |
|  | 65+ | 464(2.6) | 6(0.9) | 6(1.3) |  |
|  | *Unknown* | 117(0.7) | 2(0.3) | 2(1.7) |  |
| **Province** | City of Kigali | 6,354(35.6) | 306(48.3) | 306(4.6) | <0.001 |
|  | East | 321(1.8) | 8(1.3) | 8(2.4) |  |
|  | North | 3,477(19.5) | 124(19.6) | 124(3.4) |  |
|  | South | 2,739(15.4) | 65(10.3) | 65(2.3) |  |
|  | West | 4,935 (27.7) | 130(20.5) | 130(2.6) |  |
| **Current or past smokers** | No | 17,341(97.3) | 612(96.7) | 612(3.4) | 0.876 |
|  | Yes | 414(2.3) | 14(2.2) | 14(3.3) |  |
|  | *Unknown* | 71(0.4) | 7(1.1) | 7(9.0) |  |
| **Current or former drinker** | No | 14,056(78.9) | 74(11.7) | 74(0.5) | <0.001 |
|  | Yes | 3,691(20.7) | 552(87.2) | 552(13.0) |  |
|  | *Unknown* | 79(0.4) | 7(1.1) | 7(8.1) |  |
| **CD4(cells/mm3) at HBsAg testing** | Median (IQR) | 346 (168-564) | 322(162-544) |  | 0.225 |
|  | <350 | 2,857(16.0) | 108(17.1) | 108(3.6) |  |
|  | >=350 | 4,307(24.2) | 139(22.0) | 139(3.1) |  |
|  | *Unknown* | 10,662(59.8) | 386(61.0) | 386(3.5) |  |
| **HIV Viral load (copies/ml)** | Median (IQR) | 20(20-20) | 20(20-20) |  | 0.261 |
|  | <1000 | 11,838(66.4) | 396(62.6) | 396(3.2) |  |
|  | >=1000 | 460(2.6) | 20(3.2) | 20(4.2) |  |
|  | *Unknown* | 5,528(31.0) | 217(34.3) | 217(3.8) |  |
| **ART adherence** | Bad (<95%) | 1,050(5.9) | 17(2.7) | 17(1.6) | 0.001 |
|  | Good (>=95%) | 15,815(88.7) | 565(89.3) | 565(3.4) |  |
|  | *Unknown* | 961(5.4) | 51(8.1) | 51(5.0) |  |
| **WHO-HIV stage** | 1&2 | 13,472(75.6) | 437(69.0) | 437(3.1) | 0.002 |
|  | 3&4 | 3,737(21.0) | 163(25.8) | 163(4.2) |  |
|  | *Unknown* | 617(3.5) | 33(5.2) | 33(5.1) |  |
| **Tuberculosis** | No | 17,087(95.9) | 614(97.0) | 614(3.5) | 0.154 |
|  | Yes | 739(4.1) | 19(3.0) | 19(2.5) |  |
| **Diabetes** | No | 17,336(97.3) | 611(96.5) | 611(3.4) | 0.530 |
|  | Yes | 53(0.3) | 1(0.2) | 1(1.9) |  |
|  | *Unknown* | 437(2.5) | 21(3.3) | 21(4.6) |  |
| **TDF based regimen** | No | 4,454(25.0) | 154(24.3) | 154(3.3) | 0.880 |
|  | Yes | 12,887(72.3) | 452(71.4) | 452(3.4) |  |
|  | *Unknown* | 485(2.7) | 27(4.3) | 27(5.3) |  |
| **Time since HIV testing** | < 7 years | 6,971(39.1) | 256(40.4) | 256(3.5) | 0.284 |
|  | >= 7 years | 8,670(48.6) | 290(45.8) | 290(3.2) |  |
|  | *Unknown* | 2,185(12.3) | 87(13.7) | 87(3.8) |  |
| **Time since ART start** | < 7 years | 11,460(64.3) | 386(60.0) | 386(3.3) | 0.182 |
|  | >= 7 years | 6,071(34.1) | 229(36.2) | 229(3.6) |  |
|  | *Unknown* | 295(1.7) | 18(2.8) | 18(5.8) |  |
| * Chi-square p-value comparing HBsAg prevalence within categories | | | | | |
