# Supplementary material for: Long-term effect of chronic hepatitis B on mortality in HIV-infected persons in a differential HBV transmission setting
Source: BMC Infect Dis. 2022 May 27;22:500. doi: 10.1186/s12879-022-07477-1 (PMC9137150; doi:10.1186/s12879-022-07477-1)
Supplement: Supplementary file 2 — Additional file 2: Appendix S2. Comparing characteristics of all HIV infected screened in 2016 and the current study population. [file 12879_2022_7477_MOESM2_ESM.docx]

**Appendix S2**

The table below shows that the population characteristics in this study reflect HIV-infected individuals vaccinated and screened for HBV and hepatitis C antibodies in 2016 in Rwanda.

Table 4 Comparing characteristics of all HIV infected screened in 2016 and the current study population

|  | Umutesi et al. 2017 | | Current study | |
| --- | --- | --- | --- | --- |
|  | **Total (%)** | **Positive (95%CI)** | **Total (%)** | **Positive (95%CI)** |
| All | 114,040 | 4.3 (4.2-4.4) | 18,459 | 3.4 (3.2-3.7) |
| **Age** | **Total** | **HBsAg positive** | **Total** | **HBsAg positive** |
| <15 | 4,917 (4.4) | 2.1 (1.7-2.6) | **Not included** |  |
| 15-24 | 7,409 (6.6) | 4.0 (3.5-4.4) | 1,131 (6.2) | 2.9 (2.1-4.0) |
| 25-34 | 21,287 (19.0) | 4.2 (3.9-4.5) | 3,520 (19.2) | 3.6 (3.0-4.2) |
| 35-44 | 34,113 (30.4) | 4.8 (4.5-5.0) | 5,962 (32.5) | 3.6 (3.2-4.2) |
| 45-54 | 28,719 (25.6) | 4.5 (4.2-4.7) | 5,188 (28.3) | 3.7 (3.2-4.2) |
| 55-64 | 12,582 (11.2) | 4.0 (3.6-4.4) | 2,069 (11.3) | 2.8 (2.2-3.6) |
| ≥65 | 3,037 (2.7) | 3.9 (3.2-4.6) | 470 (2.6) | 1.3 (0.6-2.8) |
| **Sex** |  |  |  |  |
| Female | 74,141 (65.1) | 3.7 (3.5-3.8) | 12,009 (65.1) | 2.8 (2.5-3.1) |
| Male | 39,751 (34.9) | 5.4 (5.1-5.6) | 6,424 (34.8) | 4.5 (4.0-5.1) |
| **Province** |  |  |  |  |
| Kigali | 19,135 (16.8) | 5.0 (4.7-5.3) | 6,660 (36.1) | 4.6 (4.1-5.1) |
| East | 23,404 (20.5) | 5.5 (5.2-5.8) | 329 (1.8) | 2.4 (1.2-4.8) |
| North | 16,094 (14.1) | 4.2 (3.9-4.6) | 3,601 (19.5) | 3.4 (2.9-4.1) |
| South | 27,368 (24.0) | 3.6 (3.4-3.9) | 2,804 (15.2) | 2.3 (1.8-2.9) |
| West | 28,027 (24.6) | 3.5 (3.3-3.7) | 5,065 (27.4) | 2.6 (2.2-3.0) |
| **ART category** |  |  |  |  |
| On ART | 98,974 (91.6) | 4.3 (4.2-4.5) | 17,947 (97.2) | 3.6 (3.0-4.4) |
| Pre ART | 9,119 (8.4) | 4.1 (3.6-4.5) | 512* (2.8) | 3.1 (2.6-3.7) |
| **CD4 count** |  |  |  |  |
| ≤ 500 | 34,698 (41.2) | 4.9 (4.7-5.1) | 2,965 (40.0) | not provided |
| > 500 | 49,469 (58.8) | 3.7 (3.6-3.9) | 4,446 (60.0) |  |
| **HIV viral load** |  |  |  |  |
| ≤ 1,000 | 66,272 (95.1) | 3.9 (3.7-4.1) | 12,234 (96.2) | 3.2 (2.9-3.6) |
| > 1,000 | 3,406 (4.9) | 5.5 (4.6-6.4) | 480 (3.8) | 4.2 (2.7-6.4) |
| *Missing information on ART intake | |  |  |  |
