# Supplementary material for: Long-term effect of chronic hepatitis B on mortality in HIV-infected persons in a differential HBV transmission setting
Source: BMC Infect Dis. 2022 May 27;22:500. doi: 10.1186/s12879-022-07477-1 (PMC9137150; doi:10.1186/s12879-022-07477-1)
Supplement: Supplementary file 3 — Additional file 3: Appendix S3. Characteristics of individuals lost to follow up by co-infection categories. [file 12879_2022_7477_MOESM3_ESM.docx]

**Appendix S3**

The table 5 shows that female were nearly twice the total number of males lost to follow up and above ½ were young individuals. Again, Kigali City has a big number of lost to follow up, and more than three times lost while they were in the first or second WHO clinical stage. These results showed also that individuals on ART for a short time are three times more lost to follow up than those on ART for seven years or more. Considering the total number of 17,826 mono-infected individuals and 633 co-infected individuals, we find that the proportion of those lost to follow-up is 10% among the mono-infected and 12% among the co-infected.

Table 5 Characteristics of individuals lost to follow up by co-infection categories

| **Covariates** | **Category** | **Total with incomplete data (n= 1819)** | **HIV mono-infected [n= 1,740 (%)]** | **HIV-HBV co-infected [n= 78 (%)]** |
| --- | --- | --- | --- | --- |
| **Sex** | Female | 1,183 | 1,146 (96.9) | 36 (3.0) |
|  | Male | 635 | 593 (93.4) | 42 (6.6) |
|  | *Unknown* | 1 | 1 | 0 |
| **Age** | 15-24 | 241 | 234 (97.1) | 6 (2.5) |
|  | 25-34 | 583 | 557 (95.5) | 26 (4.5) |
|  | 35-44 | 509 | 483 (94.9) | 26 (5.1) |
|  | 45-54 | 299 | 284 (95.0) | 15 (5.0) |
|  | 55-64 | 135 | 131 (97.0) | 4 (3.0) |
|  | 65+ | 38 | 37 (97.4) | 1 (2.6) |
|  | *Unknown* | 14 | 14 | 0 |
| **Province** | City of Kigali | 814 | 782 (96.1) | 32 (3.9) |
|  | East | 43 | 42 (97.7) | 1 (2.3) |
|  | North | 403 | 378 (93.8) | 25 (6.2) |
|  | South | 239 | 234 (97.9) | 5 (2.1) |
|  | West | 320 | 304 (95.0) | 15 (4.7) |
| **Current or past smokers** | No | 626 | 623 (99.5) | 2 (0.3) |
|  | Yes | 977 | 901 (92.2) | 76 (7.8) |
|  | *Unknown* | *216* | 216 | 0 |
| **Current or former drinker** | No | 857 | 854 (99.6) | 2 (0.2) |
|  | Yes | 917 | 841 (91.7) | 76 (8.3) |
|  | *Unknown* | 45 | 45 | 0 |
| **CD4(cells/mm3) at HBsAg testing** | <350 | 264 | 252 (95.5) | 12 (4.5) |
|  | >=350 | 364 | 353 (97.0) | 11 (3.0) |
|  | *Unknown* | 1191 | 1135 | 55 |
| **HIV Viral load (copies/ml)** | <1000 | 1435 | 1369 (95.4) | 65 (4.5) |
|  | >=1000 | 85 | 83 (97.6) | 2 (2.4) |
|  | *Unknown* | 299 | 288 | 11 |
| **ART adherence** | Bad (<95%) | 71 | 70 (98.6) | 1 (1.4) |
|  | Good (>=95%) | 1,416 | 1,339 (94.6) | 77 (5.4) |
|  | *Unknown* | 332 | 331 | 0 |
| **WHO stage** | 1&2 | 1,136 | 1,072 (94.4) | 64 (5.6) |
|  | 3&4 | 350 | 338 (96.6) | 11 (3.1) |
|  | *Unknown* | 333 | 330 | 3 |
| **Tuberculosis** | No | 1,746 | 1,670 (95.6) | 75 (4.3) |
|  | Yes | 73 | 70 (95.9) | 3 (4.1) |
| **Diabetes** | No | 1,661 | 1,587 (95.5) | 73 (4.4) |
|  | Yes | 7 | 7 (100.0) | 0 (0.0) |
|  | *Unknown* | 151 | 146 | 5 |
| **Time since HIV testing** | < 7 years | 779 | 727 (93.3) | 52 (6.7) |
|  | >= 7 years | 596 | 581 (97.5) | 14 (2.3) |
|  | *Unknown* | 444 | 432 | 12 |
| **Time since ART start** | < 7 years | 1,188 | 1,115 (93.9) | 73 (6.1) |
|  | >= 7 years | 390 | 385 (98.7) | 4 (1.0) |
|  | *Unknown* | 241 | 240 | 1 |
